# Activation of Rho-kinase and focal adhesion kinase regulates the organization of stress fibers and focal adhesions in the central part of fibroblasts

**DOI:** 10.7717/peerj.4063

**Published:** 2017-11-15

**Authors:** Kazuo Katoh

**Affiliations:** Laboratory of Human Anatomy and Cell Biology, Faculty of Health Sciences, Tsukuba University of Technology, Tsukuba-city, Ibaraki, Japan

**Keywords:** Rho-kinase, Focal adhesion kinase, Stress fiber, Focal adhesion, Tyrosinephosphorylation

## Abstract

Specific regulation and activation of focal adhesion kinase (FAK) are thought to be important for focal adhesion formation, and activation of Rho-kinase has been suggested to play a role in determining the effects of FAK on the formation of stress fibers and focal adhesions. To clarify the role of FAK in stress fiber formation and focal adhesion organization, the author examined the formation of new stress fibers and focal adhesions by activation of Rho-kinase in FAK knockout (FAK^–/–^) fibroblasts. FAK^–/–^ cells were elliptical in shape, and showed reduced numbers of stress fibers and focal adhesions in the central part of the cells along with large focal adhesions in the peripheral regions. Activation of Rho-kinase in FAK^–/–^ cells transiently increased the actin filaments in the cell center, but these did not form typical thick stress fibers. Moreover, only plaque-like structures as the origins of newly formed focal adhesions were observed in the center of the cell. Furthermore, introduction of an exogenous GFP-labeled FAK gene into FAK^–/–^ cells resulted in increased numbers of stress fibers and focal adhesions in the center of the cells, which showed typical fibroblast morphology. These results indicated that FAK plays an important role in the formation of stress fibers and focal adhesions as well as in regulation of cell shape and morphology with the activation of Rho-kinase.

## Introduction

Actin filaments are the major components of the actomyosin contractile systems in eukaryotic cells, and function as regulators of cell movement. Activation of the Rho family of small G proteins and their downstream effector molecules (WASP/WAVE family protein and Arp2/3 complex) is accompanied by marked changes in polymerization and depolymerization of actin molecules (Uruno et al., 2001). These changes result in dynamic alterations in stress fibers, lamellipodia, and filopodia, which control cell morphology and movement.

Various physiological phenomena, including wound healing and the invasion and metastasis of cancer cells, are considered to be controlled by the actomyosin systems in many types of cells. When cultured on a glass surface, the plasma membrane of the cell begins to move in from the distal end to the leading edge. Actin cytoskeleton depolymerization deforms the morphology of the cell membrane, such that focal adhesions between the extracellular matrix and intracellular proteins move forward to the leading edge of the cell. On the other hand, stress fibers and focal adhesions are destroyed at the rear of the cell. Thus, a web-like structure is formed when the cell moving in the front portion of the cell. Such dynamic changes in the membrane structure and organelles within cells associated with cell motility require changes in cytoskeletal proteins, such as actin filaments and microtubules, which are involved in the control of membrane transport.

When moving directionally, cultured cells show highly polarized localization of receptors and adhesion molecules, such as integrin. Integrin is a focal adhesion protein that connects the extracellular matrix to the inside of the cells. Integrins are transmembrane proteins that exist as dimers of an α-chain and β-chain that act as signaling molecules between the extracellular matrix and plasma membrane in focal adhesions.

Endocytosis of integrins is actively causing stiff rather in front of the cell, although such a phenomenon at the rear of the cell are not observed. Turnover of focal adhesions by endocytosis or exocytosis of this integrin molecule involved in cell adhesion is necessary for cell movement (Paul, Jacquemet & Caswell, 2015; Ridley et al., 2003). These localization properties are controlled by focal adhesion kinase (FAK) and its associated substrates, such as members of the Src family of tyrosine kinases (Ridley et al., 2003).

Rho (Ras homology) protein is a GTPase involved in signal transduction. Activation of the Rho protein is known to regulate the organization of actin filaments in cells, including the formation of stress fibers and focal adhesions (Amano et al., 1997; Ridley & Hall, 1992). Some of these Rho associated proteins are Rho kinases (also called ROKalpha or ROCK II) (Ishizaki et al., 1996; Leung et al., 1995; Matsui et al., 1996), the myosin binding subunit of myosin phosphatase (MBS) (Kimura et al., 1996), p 140 mDia (Watanabe et al., 1997), protein kinase N (Amano et al., 1996a). Contraction of actomyosin can be regulated by kinases in two ways. The first involves phosphorylation of the MBS, then followed by the phosphorylation of the myosin light chain, result in the contraction of stress fibers in smooth muscle cells (Amano et al., 1998; Kureishi et al., 1997) and fibroblasts (Amano et al., 1998; Chihara et al., 1997).

The cell-substrate interface, which is called a focal adhesion or adhesion plaque, plays an essential role in many biological behaviors, such as cell migration, wound healing, and angiogenesis. These areas are composed of typical focal adhesion constituent proteins, such as vinculin, paxillin, talin, alpha-actinin, and integrin (Burridge, 2017; Burridge & Chrzanowska-Wodnicka, 1996). Some signal transduction proteins, such as FAK, c-Src, Rho A, and integrin, are also localized along with these constituent proteins in close association with focal adhesions. These observations suggest that the focal adhesions play roles not only in connection between the plasma membrane of the cell and substrate, but also in signal transduction from outside to the inside of the cell.

Focal adhesions recognize the boundary between the plasma membrane and suitable extracellular matrix proteins, together with the stress fibers, and such focal adhesions also determine the cell orientation and polarity (Katoh et al., 1996; Nobes & Hall, 1999; Ridley et al., 2003; Small, Anderson & Rottner, 1996). Although fibroblastic cells select specific substrates for typical cell-substrate adhesion, the mechanisms underlying initial contact with the specific substrate and the regulation of stress fibers are still unclear.

Focal adhesion and related cell scaffold proteins connect between the extracellular matrix and the cytoskeleton within the cell. Small focal adhesion-like structures are localized at the front of the leading edge of cultured fibroblasts. The signals are then transmitted to Src family kinases (SFK), such as Fyn and c-Src. FAK is then activated and undergoes autophosphorylation, and a phosphorylation of Paxillin and Cas is accelerated, and elements in the SH2 domain (Crk, Nck and Grb2) gather there, and then a big complex of the protein of well-developed focal adhesions are formed (Brunton, MacPherson & Frame, 2004; Calalb, Polte & Hanks, 1995; Parsons & Parsons, 2004). The fine filamentous actin that made the stress fibers in fibroblastic cells, which is a starting point, is formed to signal to a tyrosine phosphorylation of cortactin which controls polymerizing of actin and transfers a signal down to a Rho family G protein. As a result, the cell shows stable attachment to the substrate, and cells begin to migrate on the substrate (Evers et al., 2000; Ridley et al., 2003; Riento & Ridley, 2003). Cell migration requires continual formation and destruction of the focal adhesions.

FAK is a cytoplasmic tyrosine kinase that plays an important role in the integrin-mediated signal transduction pathway (Parsons, 2003; Schlaepfer & Mitra, 2004; Schlaepfer, Mitra & Ilic, 2004). Integrin-mediated cell adhesion leads to FAK activation and autophosphorylation in various cell types. Activated FAK is associated with several Src homology domain-containing signaling molecules, including the Src family kinases, the p85 subunit of PI3K, phospholipase C, and Grb 7 (Parsons, 2003). FAK binding to Src family kinases leads to phosphorylation of several other substrates, including FAK (Schaller & Parsons, 1994), p130Cas (Vuori et al., 1996). The interaction of FAK with these signaling molecules has been shown to induce several downstream signaling pathways that regulate cell spreading and migration, cell survival, and cell cycle progression (Parsons, 2003; Schlaepfer & Mitra, 2004).

The regulation of FAK and Rho-kinase activities is well characterized; it is not easy to determine the cooperative roles of FAK and Rho-kinase in the formation of stress fibers and focal adhesions. Local accumulation and activation of FAK is thought to be important for the formation and destruction of focal adhesions, and FAK is expected to be involved in the generation of stress fibers and focal adhesions by Rho-kinase activity. The author used FAK-knockout fibroblasts (FAK^−∕−^) to clarify the function of FAK at the time of stress fiber and focal adhesion formation, to examine the stress due to Rho-kinase activation and its associated novel formation of stress fibers and focal adhesions. These observations suggested that with the known involvement of Rho in the regulation of local adhesions, changes in Rho activity may be responsible for the abnormal behavior of FAK null mutant cells. The control of Rho-kinase changes in the absence of FAK, and these changes in Rho-kinase activity may account for some of the influence of FAK on the cytoskeleton.

## Methods

### Cell culture

Fibroblasts (NIH3T3) were cultured in a 1:1 mixture of Dulbecco’s modified Eagle’s medium and nutrient mixture (F-12; Gibco, Grand Island, NY, USA), pH 7.4, containing 50 units/mL of penicillin, 50 µg/mL of streptomycin, and 10% fetal bovine serum (Gibco). The cells were maintained at 37 °C in a humidified 5% CO_2_ atmosphere. Cells were cultured on the glass-bottomed culture dishes (φ35 mm, Matsunami glass, Tokyo, Japan) overnight and used in this study.

FAK^−∕−^ cells derived from an embryonic (E) 8.0-day-old mouse embryo with null mutations in both the FAK gene and p53 gene were obtained from ATCC (CRL-2644; American Type Culture Collection, Manassas, VA, USA) (Ilic et al., 1995).

### Antibodies and fluorescent reagents

Monoclonal anti- alpha-actinin (Sigma, St. Louis, MO, USA), monoclonal anti-paxillin (BD Biosciences, Franklin Lakes, NJ, USA), polyclonal anti-focal adhesion kinase (FAK: Takara, Shiga, Japan), polyclonal anti-tyrosine-phosphorylated Src (pY-418) (BioSource, San Jose, CA, USA) and polyclonal anti-tyrosine-phosphorylated FAK (pY-397) antibodies (BioSource) were purchased from the sources shown. FITC-labeled phalloidin for staining of actin filaments was also purchased from Cytoskeleton (Denver, CO, USA). FITC (Cytoskeleton) and rhodamine labeled-phalloidin (Molecular Probes, Eugene, OR, USA) for staining of actin filaments were also purchased from the sources shown.

### Rho-kinase inhibitor

The Rho-kinase inhibitor, Y-27632 (Uehata et al., 1997) was purchased from Tocris (Bristol, UK). Cells were cultured on the glass bottom culture dish for overnight. Recovery experiments were performed by treating cells with Y-27632 (10 μM in culture medium) for 1 h, followed by washing with fresh medium, and the process of recovery was recorded under total internal reflection fluorescence microscopy (TIRFM; Olympus, Tokyo, Japan) as described in author’s previous study (Katoh et al., 2001a).

### Immunofluorescence microscopy

Cultured cells were fixed with 1% paraformaldehyde in PBS for 30 min and treated with 0.05% Triton X-100 in PBS for 5 min for permeabilization. The cells were then treated with 10% normal goat serum for 1 h at room temperature, and stained with monoclonal anti-FAK (1:100) (Takara, Tokyo, Japan), monoclonal anti-alpha-actinin (Sigma), anti-paxillin (BD Biosciences), polyclonal anti-tyrosine-phosphorylated FAK (pY-418; BioSource) antibody, or polyclonal anti-tyrosine-phosphorylated Src (pY-418; BioSource) for 60 min. After washing in PBS for 20 min, the fixed specimens were incubated with fluorescein-conjugated goat anti-rabbit or anti-mouse IgG. Some samples were double stained with anti-FAK. Some samples were stained with FITC (Cytoskeleton) or rhodamine-labeled phalloidin (Molecular Probes) for staining of actin filaments. Samples were then observed by TIRFM.

### GFP-FAK and GFP-FRNK expression, and total internal reflection fluorescence microscopy

The cultured cells were transfected with pTagRFP-FAK (Evorogen, Moscow, Russia) or p-EGFP-FRNK (a kind gift from Dr. C Damask, UCSF) (Ilic et al., 1998) using Tfx-50 reagent (Promega, Madison, WI, USA). Both FAK and FRNK genes were located in the C-terminal domain of the multi-cloning site of the vector of pTagRFP-C1 or pEGFP-C1. Transfected cells were selected with G-418 (Roche, Penzberg, Germany), plated on glass-bottomed culture dishes, and placed on a temperature-controlled stage at 37 °C. To clearly observe the cell-substrate interface, cells were observed by TIRFM.

### Quantitative analysis of focal adhesions numbers and area

Quantitative analysis was done using open-source Fiji image analysis software (Schindelin et al., 2012). Focal adhesion size and area were measured by Fiji in anti-paxillin stained normal fibroblasts, untreated FAK^−∕−^ cells, FAK^−∕−^ cells transfected with pTagRFP-FAK, or fibroblasts transfected with p-EGFP-FRNK. Focal adhesion images were converted from a grayscale image to a binary image with white and black values. Focal adhesion number and area were counted using Fiji Software. Statistical analysis, such as standard error and *P*- value, were performed using Microsoft Excel software (Redmond, WA, USA). When comparing means of two groups, an unpaired two-tailed Student’s *t*-test was used. All statistical tests were two-sided and a *P*-value of less than 0.05 was considered statistically significant.

## Results

### Forms of stress fibers and focal adhesions in FAK^−∕−^ fibroblasts

Stress fibers are a truly contractile apparatus, which can generate isometric tension in cells (Katoh et al., 1998). This is possible only because both end of the stress fibers are anchored to the surface of the substrate via local adhesions. The author identified two types of stress fibers: those located at the peripheral portion of the cell, called peripheral stress fibers, and those located at the central portion of the cell. Rho-kinase activity is necessary for organization of the central stress fibers. On the other hand, the activities of peripheral stress fibers were mainly affected by myosin light chain kinase (MLCK), but not Rho-kinase (Katoh et al., 2001a).

**Figure 1:**
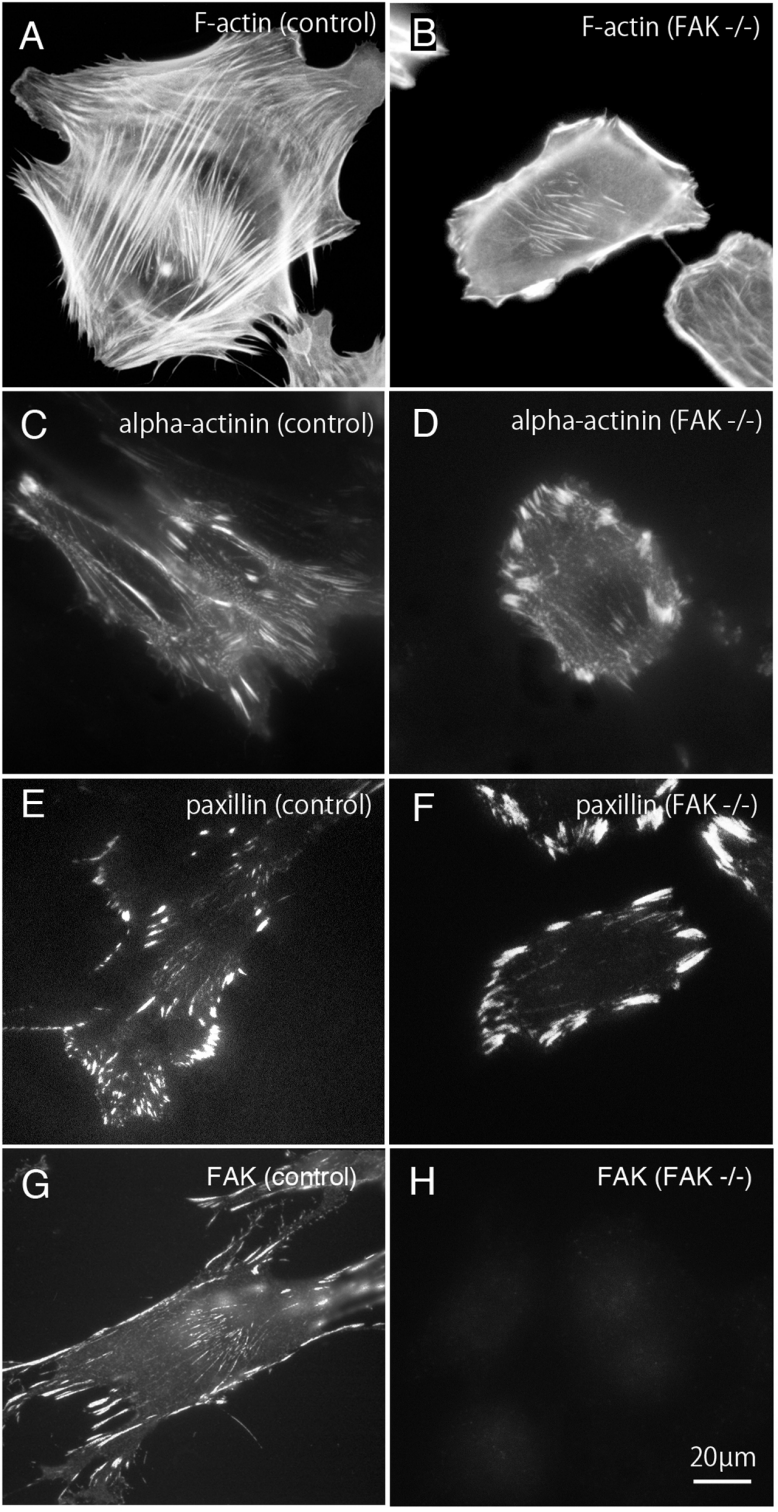
Stress fibers and focal adhesions distributed in FAK^−∕−^ fibroblasts. In normal fibroblasts, well-developed stress fibers ((A) rhodamine-labeled phalloidin staining, (C) anti alpha-actinin staining) were observed in the center of the cells. Focal adhesions were observed in both the central and peripheral part of the cell ((E) anti-paxillin staining, (G) anti-FAK staining). In FAK^−∕−^, the stress fibers were seen around the cell, but the number of stress fibers in the center was small ((B) rhodamine-labeled phalloidin staining, (D) anti-alpha-actinin staining). The focal adhesions were well developed in the peripheral part, but the number of focal adhesions in the central part was small ((F) anti-paxillin staining). No staining was detected in FAK^−∕−^ cells stained with anti-FAK antibody ((H) anti-FAK staining). Conventional epifluorescence microscopy. Bar; 20 µm

Bundles of actin filaments on the central portion of the cell seems to regulate the activation of Rho-kinase and it generates sustained tension within the cell. On the other hand, the stress fibers observed in the peripheral portion of the cell are regulated by the MLCK and generate fast and strong tension within the cell (Katoh et al., 2001b). The peripherally located stress fibers and centrally located stress fibers differ in their width. The stress fibers located at the periphery of the cell are thicker than the centrally located stress fibers. Among these, focal adhesions located at the cell periphery are larger than focal adhesions in the central portion of the cell. Together, both the central and peripheral stress fibers work together to generate a balanced condition in the cells. In this study, rhodamine-labeled phalloidin was used as stress fiber marker (Figs. 1A and 1B), and anti-paxillin antibody was used as a marker of focal adhesions (Figs. 1A and 1F). Anti-alpha actinin was detected in both the stress fibers and the focal adhesions (Figs. 1C and 1D). Anti-FAK staining was shown in normal fibroblasts (g; control) and FAK -/- cells (Fig. 1G). Well-developed stress fibers are observed in both peripheral and central portions of normal fibroblasts (Fig. 1A; F-actin, Fig. 1C; alpha-actinin or Fig. 1G; FAK). On the other hand, in FAK^−∕−^ cells, the stress fibers running peripherally are well developed, but the number of stress fibers running in the central portion is very low (Fig. 1B; alpha-actinin and Fig. 1F; paxillin). Focal adhesions of the peripheral part of the cell are significantly enlarged, but the focal adhesions in the central part are small and only a few focal adhesions were observed (Fig. 1D; alpha-actinin and Fig. 1F; paxillin). No staining with anti-FAK antibody was observed in FAK^−∕−^ cells (Fig. 1H; FAK).

### Total internal reflection fluorescence microscopy (TIRFM) imaging of FAK^−∕−^ fibroblasts with GFP-FRNK and RFP-FAK genes introduced fibroblasts

FAK is a non-receptor type protein tyrosine kinase and is involved in signaling from points of integrin condensing adhesions, thus mediating cell adhesion to the extracellular matrix. It has been reported that the signal transmitted by FAK is involved in the survival of adhesion-dependent cells and is very important for efficient cell migration in response to growth factor receptor and integrin stimulation (Mitra, Hanson & Schlaepfer, 2005; Parsons, 2003; Parsons et al., 2000; Reddig & Juliano, 2005; Schlaepfer & Mitra, 2004).

FAK knockout (FAK^−∕−^) cells showed larger focal adhesions and thicker stress fibers in the cell periphery compared to normal fibroblasts. However, there were very few focal adhesions and stress fibers in the central portion of the cell in FAK^−∕−^ fibroblasts. To confirm that FAK itself regulates the organization of centrally located focal adhesions and stress fibers, both GFP-tagged wild-type FAK gene and GFP-tagged gene encoding FAK-related non-kinase (FRNK) were introduced into the cells. FRNK genes do not have a kinase domain, and therefore FRNK gene expression acts as an inhibitor of FAK kinase regulation (Nolan, Lacoste & Parsons, 1999).

**Figure 2:**
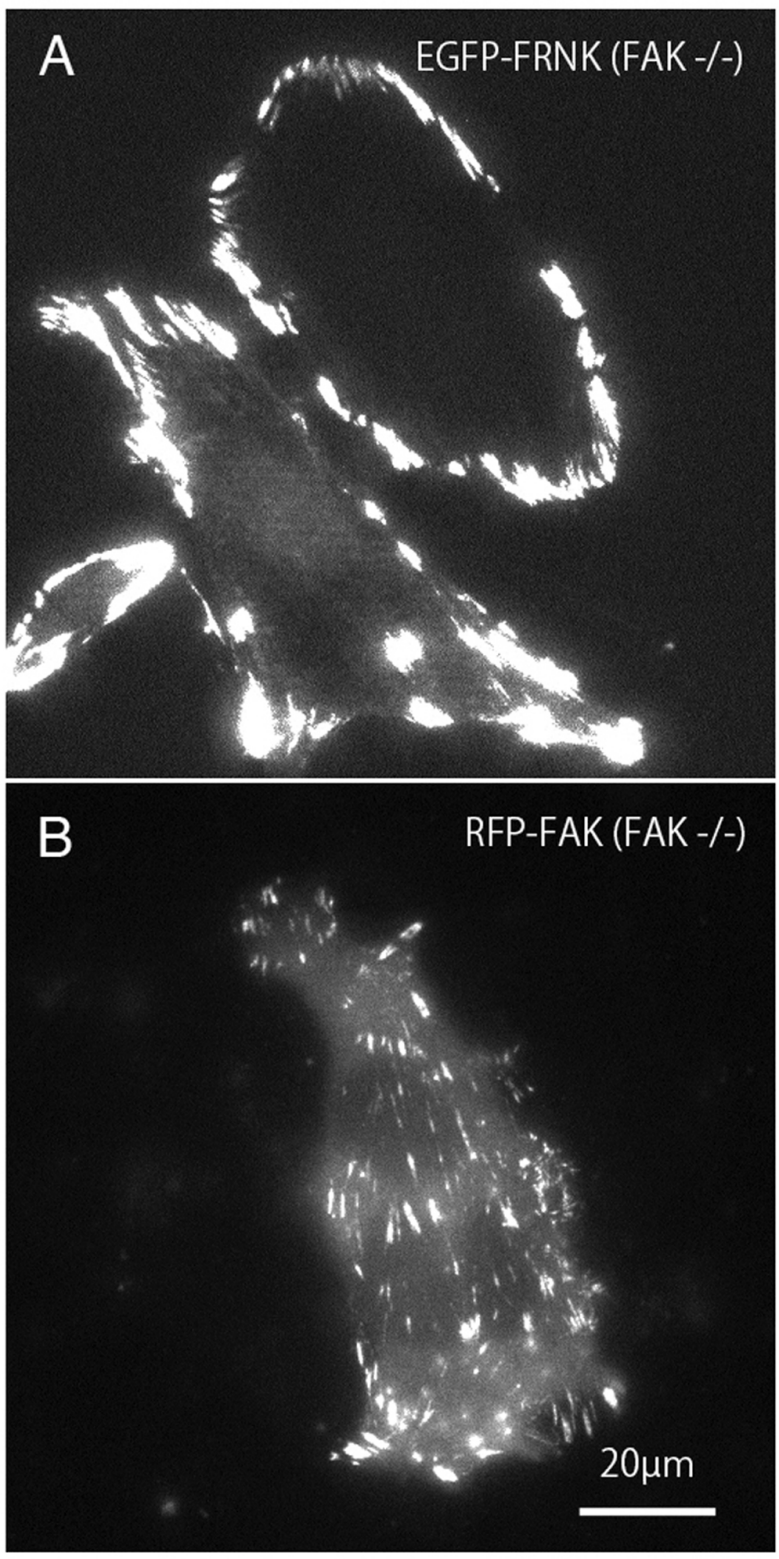
TIRFM image of FAK^−∕−^ fibroblasts transfected with GFP-FRNK and RFP-FAK. FAK^−∕−^ fibroblasts were transfected with GFP-FRNK (A) and RFP-wild-type FAK (B), and examined by TIRFM. In GFP-FRNK-transfected cells, strong expression was observed in focal adhesions in the peripheral area, but only slightly in the central area (A). The cells transfected with RFP-FAK showed spreading, the focal adhesions in the periphery were almost the same size as in normal cells, and the size and number of focal adhesions in the central part were increased on conventional epifluorescence microscopy.

Mutant GFP-FRNK (Fig. 2A) and RFP-FAK (Fig. 2B) were introduced into FAK^−∕−^ fibroblasts, and the cells were observed by total internal reflection fluorescence microscopy (TIRFM). In cells transfected with GFP-FRNK, strong expression was observed in the peripheral focal adhesions, but only slight expression was observed in the central part (Fig. 2A). Cells into which RFP-FAK had been introduced were well spread, the focal adhesions in the periphery were almost the same size as those in wild-type cells, and the size and number of focal adhesions in the central part were increased.

### Changes in focal adhesions in FAK^−∕−^ fibroblasts

FAK^−∕−^ cells showed enlarged focal adhesions at the cell periphery, and a reduction in number of focal adhesions. FRNK also regulates focal adhesions, and inhibits migration of embryonic fibroblasts, endothelial cells, and aortic smooth muscle cells (Richardson & Parsons, 1996). The changes in focal adhesion size and number in FAK^−∕−^ fibroblasts transfected with GFP-FRNK after reactivation of Rho-kinase were examined.

**Figure 3:**
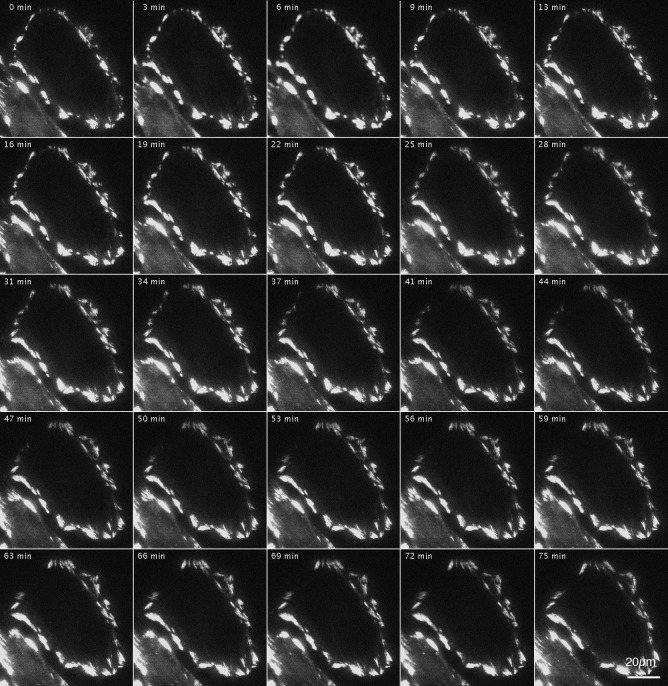
Changes in focal adhesion of FAK^−∕−^ fibroblasts transfected with GFP-FRNK by Rho-kinase inhibitor treatment. GFP-FRNK was introduced into FAK^−∕−^ fibroblasts, and the cells were then treated with the Rho-kinase inhibitor, Y-27632, for 1 h. The Rho-kinase inhibitor was then removed by washing with fresh medium, and Rho-kinase was reactivated. In cells transfected with GFP-FRNK, morphological changes in focal adhesions due to Rho-kinase activation were hardly observed. No changes were noted in the structure of plaque at the center of the cell. Time-lapse image by TIRFM.

Figure 3 shows the effects of inhibition and reactivation of Rho-kinase on focal adhesions located at the cell periphery. GFP-FRNK was introduced into FAK ^−∕−^ fibroblasts, followed by treatment with Rho-kinase inhibitor for 1 h (Fig. 3; 0 min). They were then washed with fresh medium and inhibitors were removed, which resulted in the activation of Rho-kinase. Cells were examined by time-lapse photography using TIRFM. In cells transfected with GFP-FRNK, morphological changes in focal adhesions due to Rho-kinase activation were hardly observed. No changes were observed in the structure of focal adhesions in either the peripheral or central portion of the cells (Fig. 3; 0–75 min; see also Supplemental Information 1 for live imaging).

**Figure 4:**
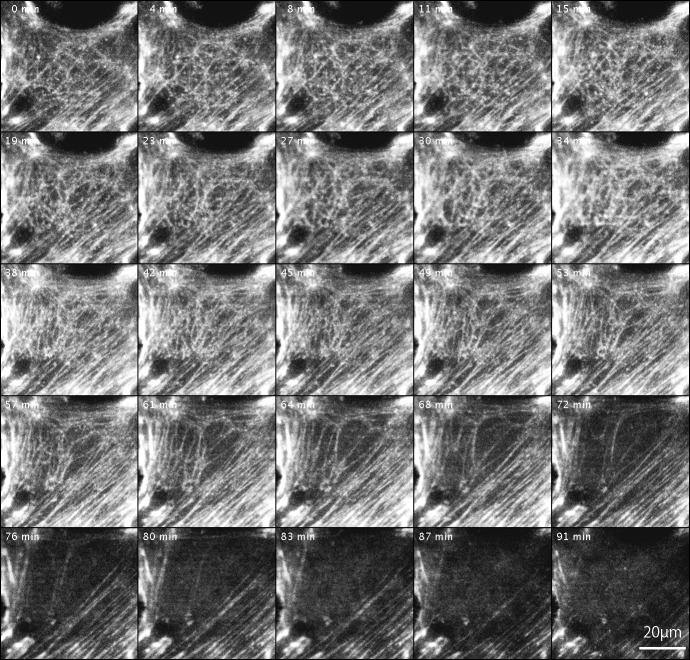
Changes in actin filaments in FAK^−∕−^ fibroblasts transfected with GFP-actin after Rho-kinase inhibitor treatment. GFP-actin was introduced into FAK^−∕−^ fibroblasts, and the cells were then treated with Rho-kinase inhibitor for 1 h. The inhibitor was removed, and Rho-kinase was reactivated. After removing the inhibitor, the reticulated actin filaments localized in the central part increased in number (0 –57 min), but did not converge and eventually disappeared (61–91 min). No new organization of stress fibers was observed. Time-lapse images by TIRFM.

### Changes in actin filaments by Rho-kinase

The changes in actin filaments by Rho-kinase in FAK^−∕−^ fibroblasts transfected with GFP-actin were also examined (Fig. 4). Cells were transfected with GFP-actin to observe stress fibers, and actin filaments at the central portion of cells were observed and recorded by TIRFM. GFP-actin was introduced into FAK^−∕−^ fibroblasts, followed by treatment with the Rho-kinase inhibitor, Y-27632, for 1 h to reduce Rho-kinase activity, and images were obtained with TIRFM (Fig. 4; 0 min). The inhibitor was then removed by washing with fresh medium, and Rho-kinase was reactivated and examined by TIRFM (Fig. 4; 4–91 min). After removing the inhibitor, the reticulated actin filaments localized in the central portion of the cell were seen to gradual increase in number within about 45 min (Fig. 4; 45 min). Interestingly, however, they did not form bundles of actin filaments, and gradually disappeared at the central portion of the cell (Fig. 4; 61–91 min). No new organization of stress fiber at the center of the cells was observed (Fig. 4; 91 min). See also Supplemental Information 2 and see also Supplemental Information 3 for live imaging.

### Novel formation of focal adhesions by activation of FAK

The focal adhesions localized at the center of wild-type cells were not formed in FAK^−∕−^ cells (Fig. 2A). Very few bundles of actin filaments were observed in the central portion of the knockout mutant cells. Transfection of RFP-labeled wild-type FAK into FAK^−∕−^ fibroblasts resulted in the formation of new focal adhesions in both the peripheral and central portions of the cell (Fig. 2B).

**Figure 5:**
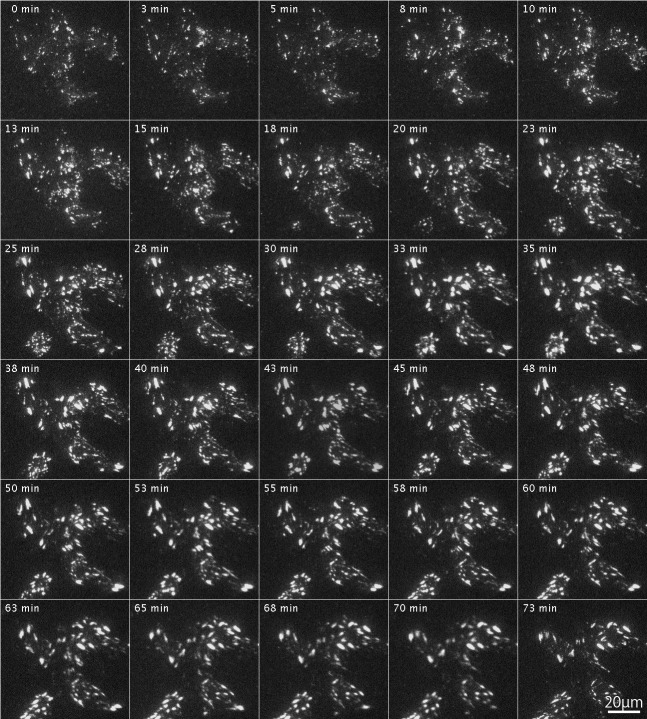
Organization of focal adhesions by FAK^−∕−^ fibroblasts transfected with RFP-FAK after Rho-kinase inhibitor treatment. RFP-FAK was introduced into FAK^−∕−^ fibroblasts, and cells were treated with Rho-kinase inhibitor for 1 h. The inhibitor was then removed, and Rho-kinase was reactivated. Small focal adhesion-like structures localized in the center of the cell increased in both number and size, and eventually became well-matured focal adhesions. Time-lapse imaging by TIRFM.

After introduction of RFP-labeled wild-type FAK (RFP-FAK) into FAK^−∕−^ fibroblasts followed by treatment with Rho-kinase inhibitor for 1 h, small focal adhesion-like structures were observed in the central portion of the cells (Fig. 5; 0 min). The inhibitor was removed by washing with fresh medium, and Rho-kinase was reactivated. The focal adhesions gradually increased in size, and well-developed focal adhesions were seen (Fig. 5; 3–73 min). These observations indicated that the wild-type FAK gene rescued FAK^−∕−^ both physically and morphologically. See also Supplemental Information 1 for live imaging.

### Tyrosine-phosphorylation of c-Src and FAK in FAK^−∕−^ fibroblasts

**Figure 6:**
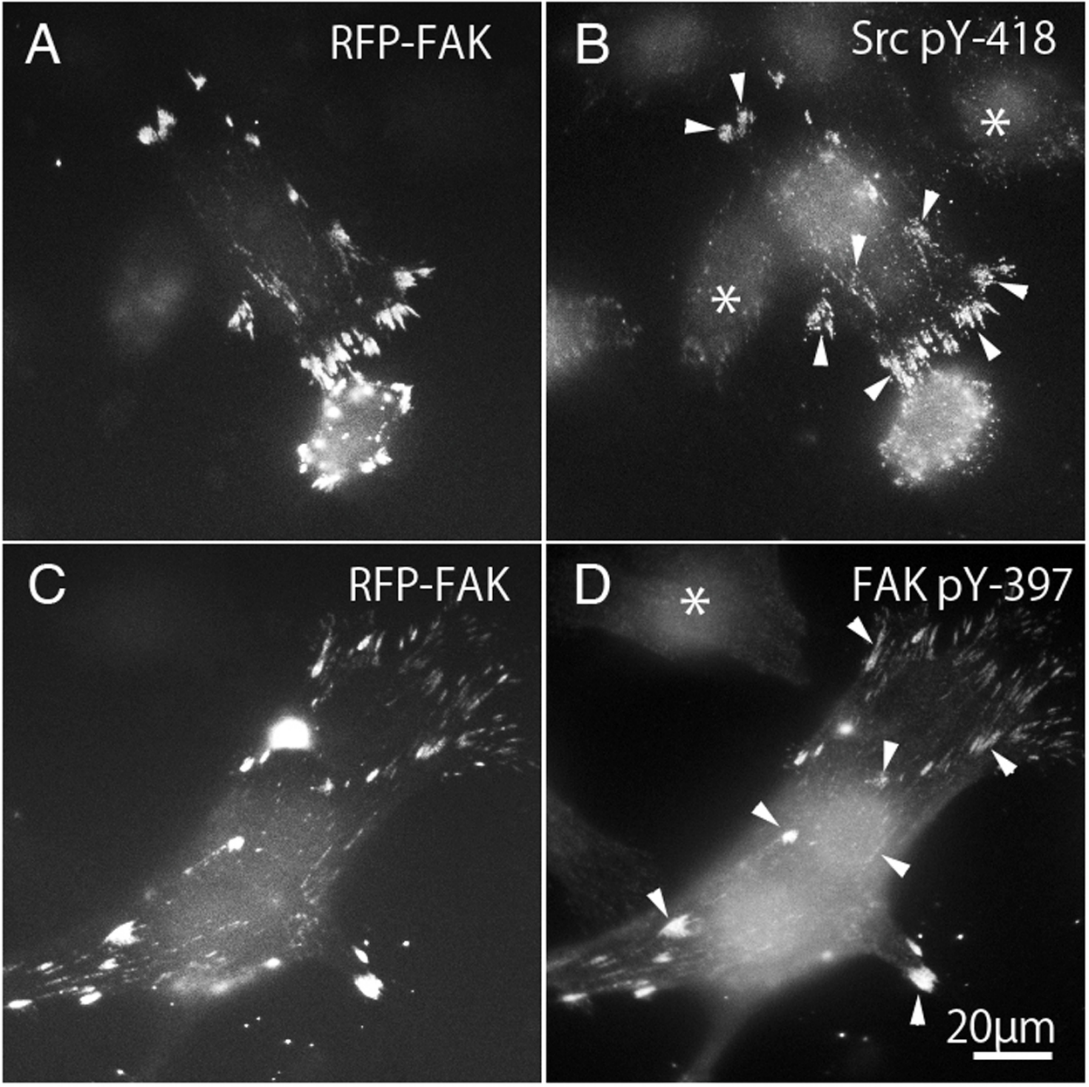
Phosphorylation of c-Src and FAK in FAK^−∕−^ fibroblasts transfected with RFP-FAK. FAK^−∕−^ fibroblasts (A and C) transfected with RFP-FAK were stained with anti-phosphorylated c-Src (pY-418) antibody (B) and anti-phosphorylated FAK (pY-397) antibody (D). The cells spread well and their morphology was similar to that of wild-type fibroblasts. RFP-FAK was found in focal adhesions in both peripheral and central parts of the cell, and tyrosine-phosphorylated c-Src (pY-418) and tyrosine-phosphorylated FAK (pY-397) were observed in focal adhesions. * Indicates the cells not transfected with RFP-FAK. Conventional epifluorescence microscopy.

Binding to Src family kinases leads to phosphorylation of FAK. Binding of integrins to the extracellular matrix causes phosphorylation of FAK at Tyr-397, which is thought to be an autophosphorylation site (Calalb, Polte & Hanks, 1995; Kornberg et al., 1991; Schaller et al., 1994). Once FAK 397 is tyrosine-phosphorylated, Tyr-397 generates high affinity binding for SH2 domain-containing proteins, such as Src family kinase (Chen & Guan, 1994; Schaller et al., 1994). On the other hand, the kinase domain of c-Src contains a tyrosine residue (pY-418) that is autophosphorylated when activated, and therefore the active form of c-Src could be detected using anti-c-Src (pY-418). Tyrosine phosphorylation of c-Src and FAK were observed in FAK^−∕−^ fibroblasts transfected with RFP-FAK (Fig. 6). FAK^−∕−^ fibroblasts (Figs. 6A and 6C) transfected with RFP-FAK were stained with anti-tyrosine phosphorylated c-Src (pY-418) antibody (Fig. 6B) and anti-tyrosine-phosphorylated FAK (pY-397) antibody (Fig. 6D). The cells spread well, and their morphology was similar to that of wild-type fibroblasts. RFP-FAK was found in focal adhesions in the peripheral and central parts of the cell, and phosphorylated c-Src and phosphorylated FAK were observed in focal adhesions. When RFP-FAK was transfected into FAK^−∕−^, anti-Src pY418 staining was observed in both the peripheral and central focal adhesions (Fig. 6B). When RFP-FAK-transfected cells were stained with anti-FAK pY-397, staining was also observed in both the peripheral and central focal adhesions (Fig. 6D). These results were similar to those observed in wild-type fibroblastic cells, indicating that the active forms of c-Src and FAK are localized in both the peripheral and central portions of the cells.

### Tyrosine-phosphorylation of c-Src and FAK in FAK^−∕−^ fibroblasts transfected with GFP-FRNK

**Figure 7:**
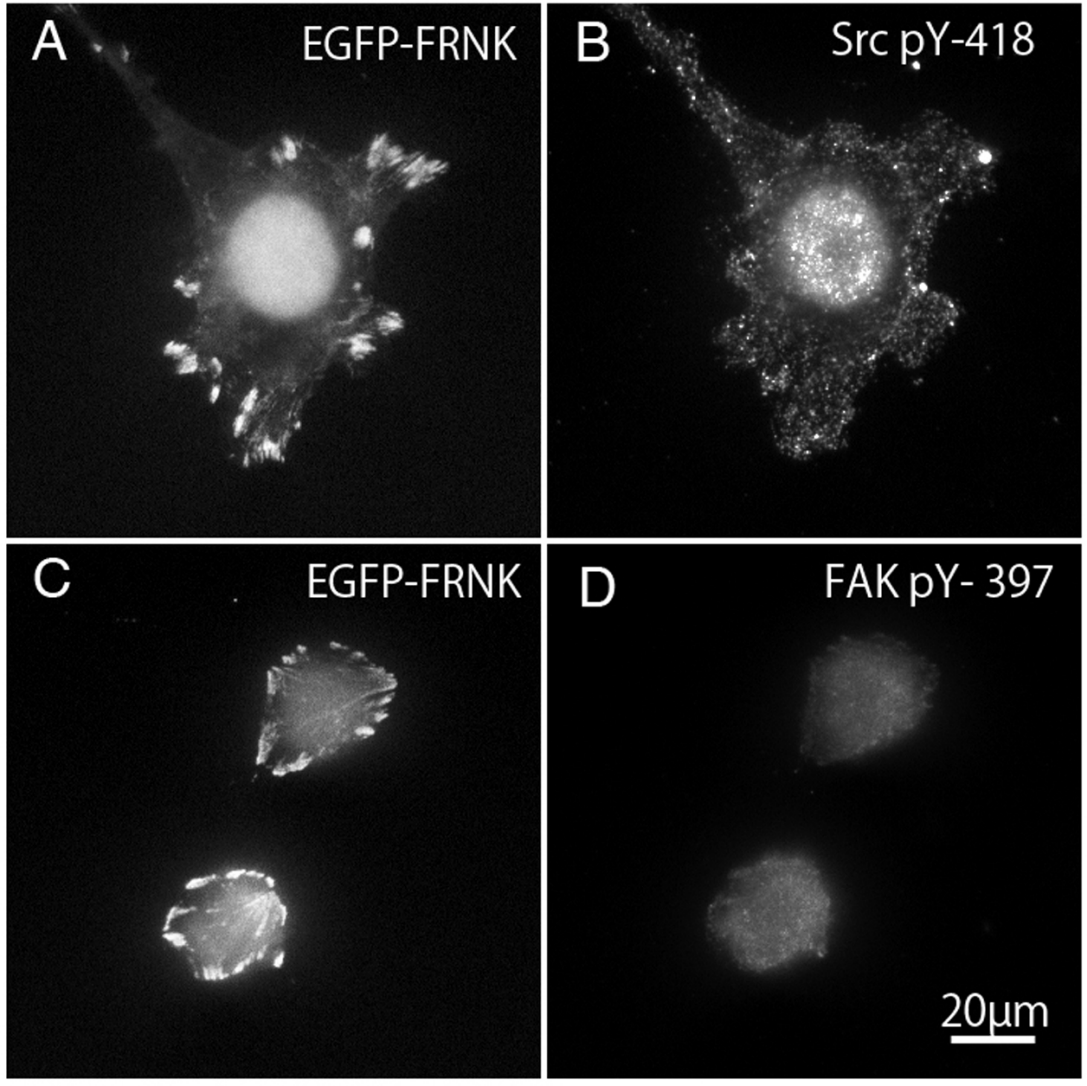
GFP-FRNK transfected cells (A and C) were stained with anti-tyrosine phosphorylated c-Src (B) and anti-tyrosine phosphorylated FAK (D).

FAK^−∕−^ fibroblasts (Figs. 7A and 7C) transfected with GFP-FRNK were stained with anti-tyrosine-phosphorylated c-Src (pY-418) antibody (Fig. 7B) and anti-tyrosine-phosphorylated FAK (pY-397) antibody (Fig. 7D). Cells transfected with GFP-FRNK did not spread and remained round in shape. Focal adhesions were located only at the cell periphery, as shown in Fig. 2A. Although GFP-FRNK was observed more often in the periphery of the cell (Figs. 7A and 7C), phosphorylated c-Src (Fig. 7B) and phosphorylated FAK (Fig. 7D) were hardly found in the focal adhesions in the cell periphery or in the center of the cell.

### Quantifative analyses of focal adhesions

**Figure 8:**
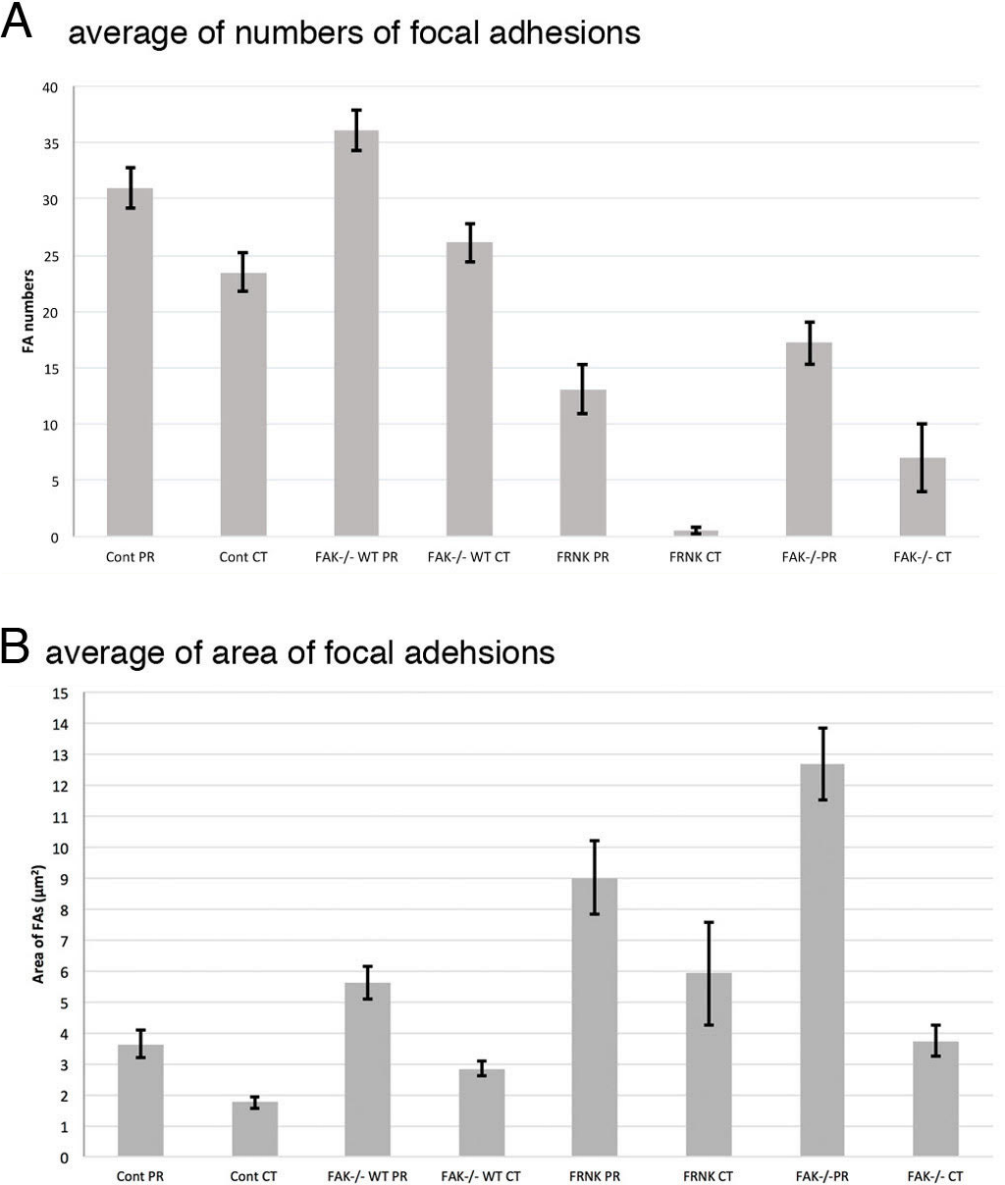
Graphical quantification of focal adhesion numbers (A) and area (B). Average of focal adhesion numbers (A) and areas were shown (B). For each experiment 10 cells were analyzed. In (B), the data were presented as an average ± standard error. Focal adhesion numbers and area were measured using Fiji software. Error bars represent standard error of the mean. *P*-value < 0.01. Cont PR, focal adehsion located at the cell periphery in normal fibroblast stained with anti-paxillin. Cont CT, focal adehsions located at the cell center in normal fibroblast stained with anti-paxillin. FAK^−∕−^ WT PR, focal adhesions located at the cell periphery in FAK^−∕−^ cells transfected with wild type RFP-FAK gene. FAK^−∕−^ WT CT, focal adhesions located at the cell center in FAK^−∕−^ cells transfected with wild type RFP- FAK gene. FRNK PR, focal adhesions located at the cell periphery in fibroblast transfected with GFP- FRNK gene. FRNK CT, focal adhesions located at the cell center in fibroblast transfected with GFP- FRNK gene. FAK^−∕−^ PR, focal adhesions at the cell periphery in FAK^−∕−^ cells. FAK^−∕−^CT, focal adhesions at the cell center in FAK^−∕−^ cells.

Summary of the average of the number and the area of focal adhesions in this study was shown in Table 1. Graphical quantification of numbers of focal adhesion and area measured from the image of immunofluorescent microscopy were also shown in Fig. 8. Averaged number of focal adhesion in both the peripheral and central portion of the fibroblast stained with anti-paxillin antibody were 31.0 ± 1.80 (standard error) and 23.5 ± 1.70, respectively. In untreated FAK^−∕−^ cells, averaged numbers of focal adhesion in peripheral was 18.2 ± 1.88 and central stained with anti-paxillin antibody was 7.0 ± 3.04. When FAK^−∕−^ cells were transfected with wild-type FAK gene, averaged numbers of focal adhesions at the cell periphery and center were 36.1 ± 6.23 and 26.1 ± 3.20, respectively. Moreover, when fibroblasts were transfected with GFP-FRNK, averaged area of focal adhesion in the peripheral and central portion of the cell were 3.64 ± 0.44 μm^2^ and 1.75 ± 0.19 μm^2^, respectively. These quantification analyses strongly support the immunofluorescent microscopy observations mentioned above.

**Table 1:** Summary of the number (A) and the area (B) of focal adhesions. Average of the number (A) and the area (B) of focal adhesions were shown. Control fibroblasts were normal fibroblast stained with anti-paxillin antibody. FAK^−∕−^ +WT FAK gene was FAK^−∕−^ cells transfected with wildtype RFP- FAK gene. Fibroblast + FRNK gene was fibroblasts transfected with GFP- FRNK gene. FAK^−∕−^ cells were untreated FAK^−∕−^ cells. Number of FAs at peripheral (or center) was an averaged number of focal adhesions located at the cell peripheral (or center). Area of FAs at peripheral (or center) was averaged area of focal adhesions located at the cell periphery (or center). When GFP-FRNK was introduced into FAK^−∕−^ cells, the quantitative analysis was excluded, because there were no significant changes in the cell morphology or in the formation of stress fibers and focal adhesions. ± represents standard error of the mean.

(A)
	Number of FAs at peripheral	Number of FAs at center
Control fibroblasts	31.0 ± 1.80	23.5 ± 1.70
FAK^−∕−^ +WT FAK gene	36.1 ± 6.23	26.1 ± 3.20
Fibroblast + FRNK gene	13.1 ± 2.17	0.55 ± 0.29
FAK^−∕−^ cells	18.2 ± 1.88	7.0 ± 3.04

(B)
	Area of FAs at peripheral	Area of FAs at center
Control fibroblasts	5.60 ± 0.53 μm^2^	2.84 ± 0.24 μm^2^
FAK^−∕−^ +WT FAK gene	3.64 ± 0.44 μm^2^	1.75 ± 0.19 μm^2^
Fibroblast + FRNK gene	9.01 ± 1.19 μm^2^	5.92 ± 1.67 μm^2^
FAK^−∕−^ cells	12.67 ± 1.15 μm^2^	3.77 ± 0.51 μm^2^

## Discussion

Tyrosine-phosphorylation of certain types of protein involved in signal transduction mechanisms in cells occurs when these proteins are activated or inactivated. The level of tyrosine-phosphorylation associated with activation or inactivation of tyrosine-phosphorylation reflects the local levels of signal transduction activity. In cells in culture, it is well known that the phosphotyrosine-proteins are highly accumulated at focal adhesions, reflecting the highly specific area of signal transduction.

Expression of the non-kinase domain of FAK, FRNK, in normal fibroblasts seems to regulate the organization of the central but not peripheral stress fibers. When GFP-FRNK, which acts as an inhibitor of FAK, significantly reduces the number and size of central stress fibers and focal adhesions in fibroblasts. Previous studies performed in author’s laboratory indicated that Rho-kinase-dependent reconstitution of stress fibers occurs along small focal adhesion-like structures located in the center of adherent fibroblasts. The accumulation and bundling of actin filaments initially occur along small focal adhesion-like structures (Katoh, Kano & Ookawara, 2007). The initial accumulation of focal adhesion-like structures seems to be regulated by the activation of Rho-kinase. Moreover, the organization of focal adhesion-like structures in the central portion of the fibroblast precedes the organization of stress fibers (Katoh, Kano & Ookawara, 2007). The results of the present study indicated that the organization of focal adhesion-like structures preceding the organization of newly forming stress fibers is tightly regulated by the activation of FAK, together with the activation of Rho-kinase. The mechanisms underlying the co-regulation of Rho-kinase activation and the activation of FAK in organization of focal adhesions and associated stress fibers have not yet been elucidated.

### Rho GTPases control the association of actin filaments with the plasma membrane

Recently, it was reported that the Rho GTPases control the association of actin filaments with the plasma membrane. The GTP-binding proteins belonging to the Ras superfamily, which regulate filamentous actin organization, are closely associated with the plasma membrane. Rho activation causes direct or indirect phosphorylation of myosin light chain, resulting in stress fiber assembly. Both ends of the stress fibers are attached to the focal adhesions, and phosphorylation of myosin light chain generates diametral tension in the cell (Katoh et al., 1998). On the other hand, activation of Rac induces the organization of lamellipodia along the leading edge of the cell.

### FAK^−∕−^ cells showed an elliptical shape

FAK^−∕−^ cells were elliptical in shape with reduced numbers of stress fibers and focal adhesions in the central part of the cell and the formation of a large focal adhesion in the peripheral part. Activation of Rho-kinase in FAK^−∕−^ cells transiently increased the number of actin filaments in the cell center, but they did not form typical stress fibers, and gradually disappeared over time. Furthermore, when the full-length FAK gene labeled with RFP was introduced into FAK^−∕−^ cells, the cells spread, the numbers of stress fibers and focal adhesions localized in the center of the cell were increased, and the cells showed the typical fibroblast morphology. Moreover, phosphorylated FAK (pY-397) and phosphorylated c-Src (pY-418) were localized in focal adhesions. On the other hand, when GFP-FRNK was introduced into FAK^−∕−^ cells, there were no changes in the cell morphology or in the formation of stress fibers and focal adhesions at the center of the cell. These results indicated that FAK and c-Src play important roles in the formation of novel stress fibers and focal adhesions accompanying the activation of Rho-kinase and in the maintenance of cell morphology.

The kinase domain of c-Src includes a tyrosine residue (Y418) that undergoes autophosphorylation on activation. Another region of c-Src close to the C-terminus contains a tyrosine residue (Y527) that is related to the regulation of activity. In the resting cell, most of Y527 in c-Src is phosphorylated and it has a compact structure with low activity due to binding with its own SH2 domain (Xu, Harrison & Eck, 1997; Young et al., 2001). When the phosphorylated Y527 residue of c-Src is dephosphorylated, the intramolecular bond is broken resulting in a change to a flexible structure, and another protein is bound to the SH2 and SH3 domains that have become free and the function is exhibited. Thus, c-Src activity and function can be controlled by tyrosine-phosphorylation and structural changes due to binding between proteins inside and outside of the molecule. The phosphorylation of Y527 in c-Src is mediated by another tyrosine kinase, C-terminal Src kinase (Csk) (MacAuley et al., 1993; Nada et al., 1991; Nada et al., 1993).

In fibroblasts, when Rho-kinase is activated intracellularly, a new small plaque-like structure is formed on the basal plane of the cell, which gradually becomes larger and changes to a typical focal adhesion structure. That is, due to the action of Rho-kinase, the plaque-like structure as the origin of the focal adhesion is formed and actin fibers newly accumulate and increase in number as the starting point and form stress fibers at the central portion of the cell (Katoh, Kano & Ookawara, 2007). In addition, on treatment with a myosin inhibitor, only the focal adhesion-like structure grew within the cells, and formation of stress fibers was not observed. These observations strongly suggested that Rho-kinase plays an important role not only in the formation of stress fibers but also in the regulation of focal adhesion formation (Katoh, Kano & Ookawara, 2007; Katoh, Kano & Ookawara, 2008).

FAK activation involves integrin receptors gathered and binding to extracellular matrix (ECM) proteins, which may include FAK dimerization (Lee et al., 1992). This involves FAK autophosphorylation of Y397, binding of Src-family kinase to phosphorylated sites, Src-mediated phosphorylation of the FAK kinase domain activation loop (Y576/577), resulting in active FAK-Src complex formation (Roy-Luzarraga & Hodivala-Dilke, 2016; Sulzmaier, Jean & Schlaepfer, 2014; Yoon et al., 2015). Inactivation of FAK using GFP-FRNK was found here to enhance peripherally located stress fibers and focal adhesions, but no such effect was observed in the central portion of the cell, as observed in FAK^−∕−^cells. Rho-kinase activation in FAK^−∕−^ cells was accompanied by increases in the number of actin filaments located in the central portion of the cell, but they did not form typical stress fibers (Fig. 4). This observation strongly suggested that both FAK and Rho-kinase activation are needed to organize the central stress fibers in fibroblastic cells. Central stress fibers and focal adhesions are expected to generate sustained tension within the fibroblast, and thus the organization of central stress fibers seems to control fine-tuned tension within the cell.

This study indicated that FAK knockout cells (FAK^−∕−^) showed an elliptical shape, decreased numbers of stress fibers and focal adhesions in the central part of the cell, and the formation of a large focal adhesion in the peripheral part of the cell. Activation of Rho-kinase in FAK^−∕−^ increased the number of thin actin filaments in the central portion of the cell, but these did not become typical stress fibers, and the actin filaments gradually disappeared at the center. Furthermore, when the full-length wild-type FAK gene labeled with RFP was introduced into FAK^−∕−^, the cell spreads normally and the stress fibers and focal adhesions localized in the center of the cell increased in both number and size, and the cells showed the typical fibroblast morphology. Moreover, localization of tyrosine-phosphorylated FAK (pY-397) and phosphorylated c-Src (pY-418) was clearly observed in the focal adhesions. On the other hand, when GFP-FRNK was introduced into FAK^−∕−^, there were no changes in the cell morphology or in the formation of stress fibers and focal adhesions at the center of the cell. These results indicated that FAK and c-Src play important roles in the formation of stress fibers and focal adhesions accompanying the activation of Rho-kinase, and maintenance of cell morphology.

## Conclusion

Activation of Rho-kinase in FAK^−∕−^ cells transiently increased the actin filaments in the cell center, but these did not form typical thick stress fibers. Only plaque-like structures as the origins of newly formed focal adhesions were observed in the center of the fibroblast. Introduction of an exogenous GFP-labeled FAK gene into FAK^−∕−^ cells resulted in increased numbers of stress fibers and focal adhesions in the center of the fibroblasts, which showed normal fibroblast morphology. Above results indicated that FAK plays important roles in the formation of stress fibers and focal adhesions as well as in regulation of cell shape and morphology with the activation of Rho-kinase.

##  Supplemental Information

10.7717/peerj.4063/supp-1 Supplemental Information 1 Video image of Fig. 3

10.7717/peerj.4063/supp-2 Supplemental Information 2 Video image of Figs. 4 (Low power view)

10.7717/peerj.4063/supp-3 Supplemental Information 3 Video image of Figs. 4 (high power view)

10.7717/peerj.4063/supp-4 Supplemental Information 4 Video image of Figs. 5

## Additional Information and Declarations

### Data Availability

The following information was supplied regarding data availability: The raw data has been provided as Supplemental Files.
